# Reduction of the Cytosolic Phosphoglucomutase in Arabidopsis Reveals Impact on Plant Growth, Seed and Root Development, and Carbohydrate Partitioning

**DOI:** 10.1371/journal.pone.0112468

**Published:** 2014-11-17

**Authors:** Irina Malinova, Hans-Henning Kunz, Saleh Alseekh, Karoline Herbst, Alisdair R. Fernie, Markus Gierth, Joerg Fettke

**Affiliations:** 1 Plant Physiology, University of Potsdam, Potsdam-Golm, Germany; 2 Biopolymers analytics, University of Potsdam, Potsdam-Golm, Germany; 3 Plant Physiology, Washington State University, Pullman, Washington, United States of America; 4 Max-Planck-Institute of Molecular Plant Physiology, Potsdam-Golm, Germany; 5 Department of Botany II, University of Cologne, Cologne, Germany; RIKEN Center for Sustainable Resource Science, Japan

## Abstract

Phosphoglucomutase (PGM) catalyses the interconversion of glucose 1-phosphate (G1P) and glucose 6-phosphate (G6P) and exists as plastidial (pPGM) and cytosolic (cPGM) isoforms. The plastidial isoform is essential for transitory starch synthesis in chloroplasts of leaves, whereas the cytosolic counterpart is essential for glucose phosphate partitioning and, therefore, for syntheses of sucrose and cell wall components. In Arabidopsis two cytosolic isoforms (PGM2 and PGM3) exist. Both PGM2 and PGM3 are redundant in function as single mutants reveal only small or no alterations compared to wild type with respect to plant primary metabolism. So far, there are no reports of Arabidopsis plants lacking the entire cPGM or total PGM activity, respectively. Therefore, *amiRNA* transgenic plants were generated and used for analyses of various parameters such as growth, development, and starch metabolism. The lack of the entire cPGM activity resulted in a strongly reduced growth revealed by decreased rosette fresh weight, shorter roots, and reduced seed production compared to wild type. By contrast content of starch, sucrose, maltose and cell wall components were significantly increased. The lack of both cPGM and pPGM activities in Arabidopsis resulted in dwarf growth, prematurely die off, and inability to develop a functional inflorescence. The combined results are discussed in comparison to potato, the only described mutant with lack of total PGM activity.

## Introduction

Phosphoglucomutase (PGM) catalyzes the reversible interconversion of glucose 6-phosphate (G6P) and glucose 1-phosphate (G1P). In higher plants PGM activity is verifiable in two compartments, the plastidial stroma and the cytosol. The plastidial isoform is essential for the formation of glucose 1-phosphate a substrate of ADPglucose pyrophosphorylase and, therefore, for starch synthesis. Lack of this isoform results in dramatically diminished starch levels [1], [2]. Furthermore, mutants lacking the ability to form starch displayed a higher amount of soluble sugars, like glucose and sucrose [3], [4]. The latter carbohydrate is the main transport form in higher plants and supplies non-photosynthetic tissues and organs of the plant with energy and carbon. Sucrose is formed in the light from triose-phosphates exported from the chloroplasts. During the formation of sucrose the cytosolic PGM (cPGM) is essential as it converts G6P into G1P, which is the substrate for the UDPglucose pyrophosphorylase.

Also in the dark, when the photosynthetic driven export of carbon from the chloroplast is absent, the formation of sucrose is dependent on cPGM activity [5], [6]. Furthermore, this pathway is linked to starch breakdown products. By the action of various enzymes, in most cases hydrolyzing enzymes, the transitory starch is degraded and the major carbohydrates released from the chloroplasts are glucose and maltose [5], [7], [8]. Starch derived maltose enters the cytosol via maltose exporter 1 (MEX1; [9]) and is further metabolized by disproportionating enzyme 2 (DPE2; [10], [11], [12]). DPE2 transfers one of the glucosyl residues (the non-reducing) of maltose on cytosolic heteroglycans and releases the second as free glucose. The glucosyl residues of the cytosolic heteroglycans can be released as G1P by the action of the cytosolic phosphorylase (AtPHS2; [13], [14]). However, the starch derived glucose is exported from the chloroplast via pGlcT [15], [16]. Both the exported glucose and the glucose released by the action of DPE2 are thought to be immediately converted into G6P by the action of hexokinase [5]. The cPGM controls partitioning of both sugar phosphates in the cytosol. G6P is used primarily in respiratory pathways, whereas G1P is linked to sucrose metabolism and in addition to cell wall synthesis. *Arabidopsis thaliana*, tobacco and maize contain one plastidial and two cytosolic isoforms; for potato and spinach only one plastidial and one cytosolic isoform were reported [17], [18], [19], [20], [21]. Recently, potato plants with antisense repression of cytosolic phosphoglucomutase were analyzed. These plants displayed a stunted phenotype, diminished root growth and reduced tuber yield [20]. Antisense plants were also characterized by reduced rates of photosynthesis and dramatic reduction in nucleotide level compared to the wild type [22]. Moreover, transgenic lines with altered cPGM activity revealed alterations in starch-related cytosolic heteroglycans. From these results it was concluded that elevated levels of cPGM activity favor the cytosolic phosphorylase-mediated conversion of glucosyl residues from the cytosolic heteroglycans into the cytosolic hexose-phosphate pools during starch degradation [23].

The two genes encoding cytosolic phosphoglucomutase activities in *Arabidopsis thaliana* At1g23190 (PGM 3) and At1g70730 (PGM2) [24], [17] reveal high sequence homology as well as possess similar exon/intron structures. Indeed, they encode two isoforms with 91% sequence identity at the amino acid level. Egli *et al.* [24] reported that *pgm2* and *pgm3* mutants deficient in one of the cytosolic isoforms grown under standard 12 h light/12 h dark regime displayed phenotypes similar to that of wild type. The authors suggested that under these conditions the functions of the isoforms were redundant to one another and the loss of one isoform did not affect plant metabolism. Unfortunately, the generation of double mutants was unsuccessful, as formation of homozygous seeds was prevented. Therefore, it was concluded that an absolute lack of cPGM activity compromises gametophyte development [24].

Not so long ago, transgenic potato lines with strongly decreased total PGM activities were identified. Transgenic plants were reduced in growth, tuber yield, and revealed lower levels of starch and sucrose in leaves compared to wild type [25]. Interestingly, rate of starch synthesis was similar to the wild type [26]. A possible explanation for this phenotype is a direct G1P transport over the plastidial membranes, which has been verified for both potato and Arabidopsis [27], [1].

However, until now no *A. thaliana* transgenic plants with a strong reduction of both cPGM isoforms or the simultaneous reduction of plastidial and cytosolic phosphoglucomutases have been reported. For this reason, we generated and analyzed Arabidopsis lines with *amiRNA* (artificial micro RNA) repression of both cPGMs. Furthermore, the cPGM *amiRNA* construct was introduced into *pgm1* mutants by Agrobacterium mediated transformation to explore whether a similar bypass to that observed in potato also occurred in Arabidopsis. In order to test this, the generated plants were assessed at the level of isoform specific activity as well as carbohydrate and metabolite content and phenotypic characterization of vegetative growth and propagative development. Results are discussed in the context of current understanding of the importance of the reactions catalyzed by phosphoglucomutase.

## Materials and Methods

### Plant material and growth conditions

The *pgm1* mutants were as described in [17]. The *pgm2* [SALK_068481 (AR)] and *pgm3* [SALK_023069 (AZ)] mutants were ordered from NASC. Mutants were identified by PCR amplification using the primers presented in Table S1 in File S1.

*pgm2 pgm1* and *pgm3 pgm1* were generated by crosses between individual homozygous mutants and the resulting F1 generations were allowed to self-pollinate. Double mutants were identified in the F2 generation by native PAGE and PGM activity staining (see below). For further analyses plants of F3 or F4 generation were used. Plants were grown either in 14, 10, 8 or 7 h light (110 µmol m^−2^s^−1^, 22°C; dark, 18°C, humidity 60%) or in a 12 h diurnal cycle (12 h light [110 µmol m^−2^s^−1^], 20°C; 12 h dark, 16°C, humidity 60%). For all Arabidopsis lines used, the genetic background was Col-0.

### Generation of *amiRNA* c-pgm plants

The PGM2/3-specific *amiRNA* (tctgttaagataaatgcgcct) was designed and amplified by three consecutive PCR reactions according to guidelines found at http://wmd3.weigelworld.org using the vector pRS300 as template [28] (Table S1 in File S1). The final PCR product including the cPGM-specific *amiRNA* was subcloned into the pENTR/D-TOPO vector and sequence identity was verified. Subsequently, the *amiRNA* was recombined by L/R reaction into pGWB2 [29] to obtain the binary expression plasmid *p35S:amiRNA cPGM*. The binary vector was transformed into *Argobacterium tumefaciens* strain GV3101 and used for plant transformation.

Plant transformation was performed using the floral dip method [30]. Agrobacterium strains were grown in 1 L of LB medium containing antibiotics rifampicin (100 mg/L), kanamycin (50 mg/L), gentamycin (25 mg/L), hygromycin (50 mg/L) at 28°C for 24 h. Cells were collected by centrifugation at 4,000 ***g*** for 15 min at room temperature (RT) and gently resuspended in 1 L of freshly made 5% [w/v] sucrose solution containing 0.02% [v/v] Silwet L-77 (Lehle Seeds, USA). Col-0 and *pgm1* plants (approximately four to five weeks after germination) were used for transformation. On reaching the mature stage plants were transferred to a 14 h light/10 h dark regime until mature silique stage.

### Screening of *amiRN*A plants

Dry seeds from transformed plants were collected and sterilized. Seeds were immersed in 70% [v/v] ethanol for 5 min, followed by a 20 min soaking in 2.4% [w/v] sodium hypochlorite, 0.02% [v/v] Triton X-100. Seeds were rinsed six times with sterile water and dried under sterile conditions. Seeds were screened on MS-plates with sucrose (4.3 g/L MS salt (Duchefa, Haarlem, Netherlands), 2.5 mM MES, pH 5.7 (NaOH), 1% [w/v] sucrose, 0.8% [w/v] Agar-agar) except where indicated. Selective antibiotics were added: hygromycin (50 mg/L), kanamycin (50 mg/L). Plates were placed in growth chambers and plants were germinated under 12 h light/12 h dark, except otherwise stated. Transformants with well developed leaves (four leaves stage) and roots were planted in soil and grown under standard conditions (12 h light/12 h dark). Seeds of at least four plants were harvested separately and used for generation of four plant lines *(pgm2/3 a* to *d*). Analyses were performed with the F3 to F5 generation of the respective lines.

### Phosphoglucomutase assay and PGM activity staining

Buffer-soluble proteins were extracted as described elsewhere [12]. Phosphoglucomutase activity measurement was performed as described [23]. However, in the reaction mixture soluble starch and rabbit muscle phosphorylase were omitted. Measurement was started by addition of 17.5 mM G1P to the reaction mixture. Native PAGE and PGM activity staining were performed according to Fettke *et al*. [23].

### Carbohydrate quantification

Starch was extracted and measured as described [1]. Monosaccharides, disaccharides and sugar phosphates were determined according to Stitt *et al*. [31].

### Isolation and analysis of cell wall matrix polysaccharides

Leaf material, frozen in liquid nitrogen, was homogenized and resuspended in ice-cold 20% [v/v] ethanol, mixed thoroughly, and centrifuged for 10 min at 20,000 ***g*** (4°C). Pellets were washed with 20% [v/v] ethanol two times, finally resuspended in 70% [v/v] ethanol and centrifuged (as above). Subsequently, pellets were resuspended in chloroform/methanol (1:1 [v/v]) and incubated for 20 min under continuous stirring followed by centrifugation (as above). The resulting pellets were completely destained by washing with acetone followed by water. Then pellets were resolved in 0.1 M sodium acetate buffer (pH 5.0) and incubated for 20 min at 80°C. The suspension was cooled to RT and residual starch was removed by treatment with 25 U of α-amylase (from *Basillus sp*. Typ II-A, Sigma-Aldrich, Germany) and 7 U pullulanase (from *Klebsiella planticola*, Macerozyme, Ireland) as described elsewhere [32]. The residual pellet was washed at least five times with water and subjected to TFA hydrolysis (2 M final concentration) for 3 h at 100°C. After that samples were centrifuged and the supernatants were collected. Pellets were washed two times with water and supernatants pooled together. Collected supernatant represents matrix polysaccharides of the cell wall. Following lyophilization, samples were dissolved in water and monomer content was estimated [33] (glucose was used as a standard). Aliquots were subjected to HPAEC-PAD for monosaccharide separation (as described elsewhere [12]).

### Isolation and quantification of crystalline cellulose

Residual pellets from cell wall matrix isolation were subjected to hydrolysis in Updegraff reagent (8:1:2 of concentrated acetic acid:concentrated nitric acid:water) [34] for 30 min at 100°C. Crystalline cellulose was separated, completely hydrolyzed into glucose, and determined as described elsewhere [35].

### Metabolic Profiling

For GC-MS analyses, Col-0 and transgenic lines were grown in 12 h light/12 h dark regime and harvested at the end of the light and at the end of the dark. Plants were five-week-old. Leaves from several plants per line were pooled together and processed as previously described [36].

### Trypan blue staining

Trypan blue (Sigma-Aldrich, Germany) staining was performed as described [37]. Leaves were boiled 1 min at 100°C with lactophenol-trypan blue solution (10 mL lactic acid, 10 mL glycerol, 10 g phenol, 10 mL 0.1% [w/v] trypan blue solution) and decolorized with chloral hydrate (2.5 g mL^−1^ distilled water) overnight.

### Statistical analysis

Statistical analysis (Student’s t-test [two-sided]) was performed using MS Excel 2010 (Microsoft Corporation, Washington, USA).

## Results

### Elimination of one cPGM isoform in Arabidopsis has no significant effect on starch metabolism

In native PAGE the total PGM activity was resolved in three distinct bands of activity, the fastest moving band represented the plastidial PGM (PGM1), whereas the slowest moving band represented PGM3 (At1g23190) and the intermediate band PGM2 (At1g70730). Both PGM2 and PGM3 are cytosolic isoforms [23], [24]. The localization of the three isoforms was further confirmed by non-aqueous fractionation [38]. All three isoforms were detected in various organs (Fig. S1A in File S1). PGM activity was analyzed in leaves of different Arabidopsis accessions (Fig. S1B in File S1). Results indicate a wide diversity of cytosolic PGM isoforms. Consistent with previously published data [24], Cvi-0 was the single accession which displayed only one cytosolic isoform.

Two mutants lacking an isoform of cytosolic PGM (*pgm2, pgm3*) were previously analyzed [24]. No substantial differences compared to the wild type were observed even when various parameters like starch and soluble sugar content as well as root and shoot growth were examined. However, we here generated independent homozygous T-DNA mutant lines. The total reduction in PGM activity was determined to be 23% in *pgm3* plants and 35% in *pgm2* plants compared to control Col-0. These results were consistent with the PGM activity staining analysis (Fig. S1B in File S1), since the PGM2 band had a higher intensity than PGM3.

Additionally, PGM2 and PGM3 proteins from *A. thaliana* have previously been cloned and expressed in *Escherichia coli* and the recombinant proteins were analyzed for substrate specificity and affinity. However, no differences between PGM2 and PGM3 were observed [39].

In order to analyze the influence of different growth conditions on *pgm2* and *pgm3* mutants, plants were cultivated under various light/dark conditions (light phase: 7 h, 8 h, 10 h or 14 h). Still both mutants revealed a similar growth phenotype (data not shown) and starch content compared to the Col-0. The *pgm2* plants displayed an increased level of sucrose under different growth conditions but this was not observed for *pgm3* (Table 1). Most likely, PGM2 has a higher impact on glucose-phosphate turnover. However, no significant differences in steady-state levels of sugar phosphate contents (F6P, G1P, G6P) were observed (data not shown).

**Table 1:** Carbohydrate content.

carbohydrate	growth photoperiod [h light]	Col-0	*pgm3*	*pgm2*
starch [mg/g FW]	7 h	6.1±0.3	5.4±0.6	6.1±0.3
	8 h	9.2±0.4	7.7±0.3	9.4±0.2
	10 h	6.9±0.4	5.0±0.1	6.1±0.2
	14 h	7.0±0.7	6.7±0.6	6.4±0.6
sucrose content [µmol/g FW]	7 h	1.55±0.11	1.45±0.03	1.81±0.03*
	8 h	1.17±0.04	1.04±0.05	1.87±0.33*
	10 h	1.86±0.08	1.96±0.09	2.71±0.05*
	14 h	2.58±0.19	2.46±0.21	2.90±0.03**

Leaves were harvested one hour before beginning of the dark phase. Values are means of four replicates representing a mix of 7–10 plants ± SD. Asterisks denote the significance levels as comparing mutants to Co1-0 : * *p*≤0.01;** *p*≤0.05.

As the cytosolic pools of sugar phosphates are linked to starch metabolism via the action of two transglucosidases (DPE2 and AtPHS2), the activity of both enzymes and the composition of soluble heteroglycans (SHG_L_) were analyzed. However, neither differences in enzyme activities nor composition of SHG_L_ were observed (Fig. S2 in File S1).

Thus, it seems likely that PGM2 and PGM3 could substitute for one another since the residual PGM activity in either mutant is relatively abundant.

### Simultaneous reduction of PGM2 and PGM3 activities affect plants growth and carbohydrate partitioning

**Figure 1:**
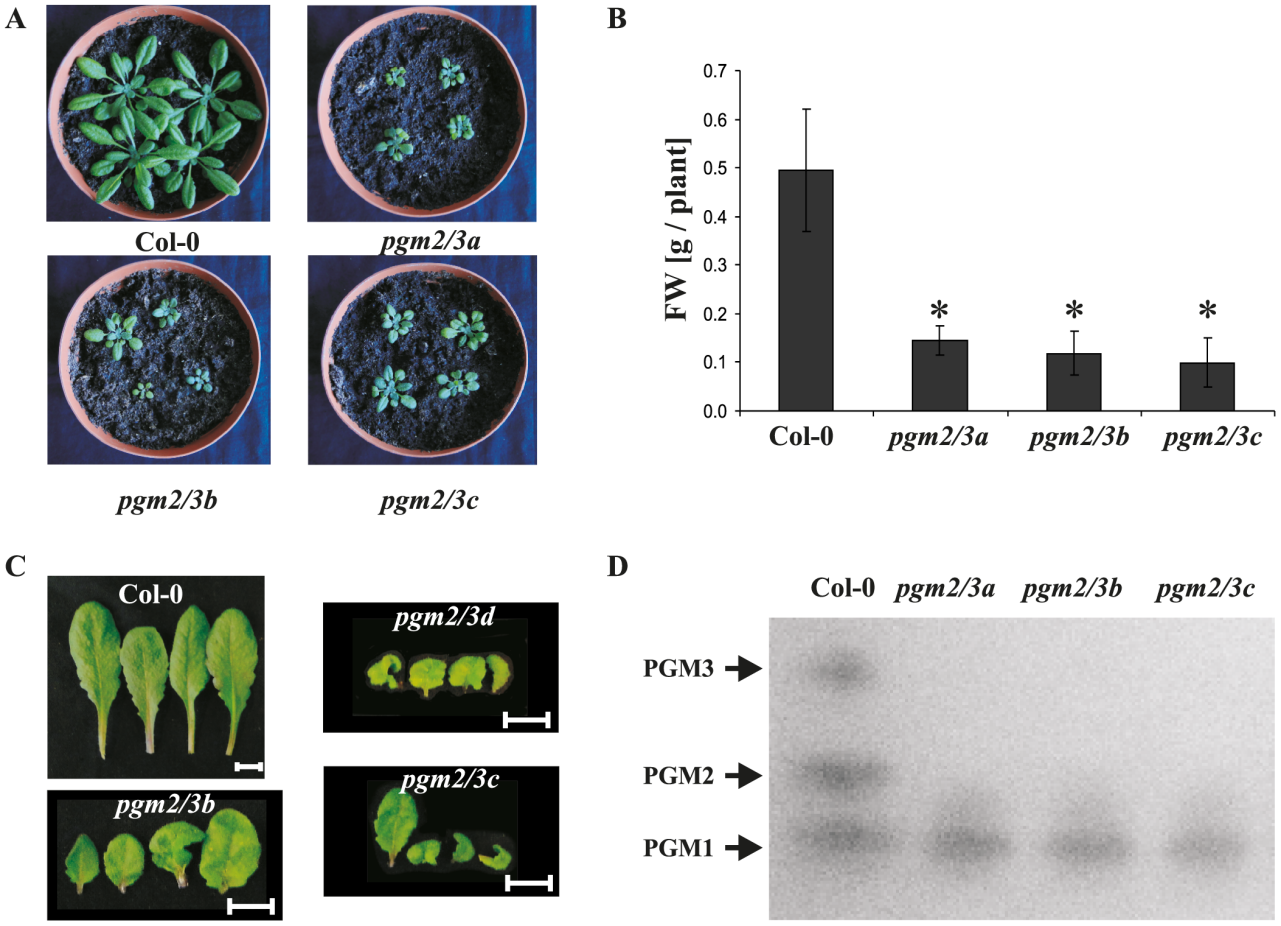
Phenotype of Col-0 and *pgm2/3* plants in 12 h light/12 h dark regime. A, Growth phenotypes. Photographs were taken of six-week-old plants. Bar = 1 cm. B, Fresh weight of plant rosettes. Values are means ± SD (n = 29−30). Plants were harvested after five weeks. Asterisks indicate significant difference from Col-0 (Student Test, P≤0.01). C, Leaf form from Col-0 and transgenic plants. Leaves were harvested from the middle of rosettes from six-week-old plants. Bar = 1 cm. D, Phosphoglucomutase activity in Col-0 and *pgm2/3* plants. Crude extracts were subjected to native PAGE and subsequent PGM activity staining. Separation gel was 7.5% [T] and 25 µg protein was loaded per lane.

Given that single *pgm2* and *pgm3* mutants do not reveal significant changes in e.g. starch metabolism, generation of double mutants is essential to clarify the role of cPGM for plant metabolism. An *amiRNA* cPGM construct was therefore transformed into Col-0 plants and four independent lines were generated. Transgenic *pgm2/3* lines were strongly retarded in growth and revealed diminished fresh weight compared to Col-0 (Fig. 1A–B). Additionally, *pgm2/3* leaves revealed small and abnormally curled leaves (Fig. 1C) and slightly elevated chlorophyll levels (Table S2 in File S1). Protein crude extracts of Col-0 and *pgm2/3* leaves were subjected to native PAGE and PGM activity staining (Fig. 1D). In all *pgm2/3* lines the two bands of cPGM activity were below the limit of detection (cPGM activity was not observed, even if 75 µg of protein crude extracts were loaded on the gel; data not shown). In addition, PGM activities in protein crude extracts were measured (Fig. S3A in File S1). In all three transgenic lines a strong reduction in total PGM activity was observed (residual activity 30–34%, [wt = 100%]). Furthermore, analyses of gene expression revealed that PGM2 and PGM3 were strongly down-regulated in *pgm2/3* lines. In contrast PGM1 was somewhat up-regulated (Fig. S3B in File S1).

However, transgenic *pgm2/3* plants grown under prolonged day conditions (14 h light/10 h dark) revealed similar results with transgenic plants being significantly smaller than Col-0, but larger as compared to the 12 h light/12 h dark grown plants (Fig. S3C in File S1).

**Figure 2:**
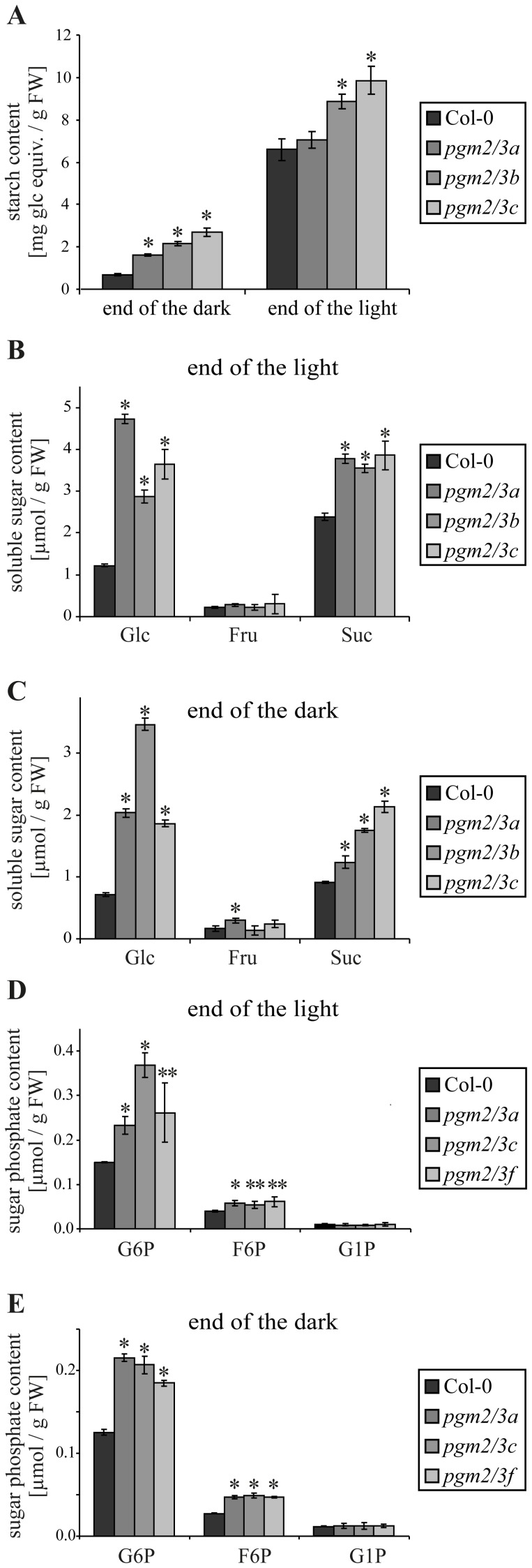
Carbohydrate analysis of Col-0 and *pgm2/3* plants. A**–**E, Plants were grown under 12 h light/12 h dark conditions and after five weeks 7–8 plants were collected and homogenized per line. Values are means of four technical replicates (A–C), and three technical parallels (D–E) ± SD, respectively. A, Starch content. B–C, Soluble sugar content. D–E, Sugar phosphate content. Asterisks denote the significance levels comparing *pgm2/3* mutants to Co1-0: * *p*≤0.01;** *p*≤0.05.

With respect to metabolites all *pgm2/3* lines showed increased starch content at the end of the dark phase compared to Col-0 (Fig. 2A). The increased starch content was also detected at the end of the light phase except for *pgm2/3a.* Similarly, starch content was significantly increased in *pgm2/3* lines compared to Col-0 when grown in 14 h light/10 h dark regime (data not shown). Transgenic *pgm2/3* lines displayed increased levels of glucose and sucrose on a fresh weight basis. In contrast the amount of fructose was comparable in the transgenic lines and Col-0 (Fig. 2B–C). Similar results were also obtained, if metabolite content was evaluated on a dry weight basis (data not shown).

**Figure 3:**
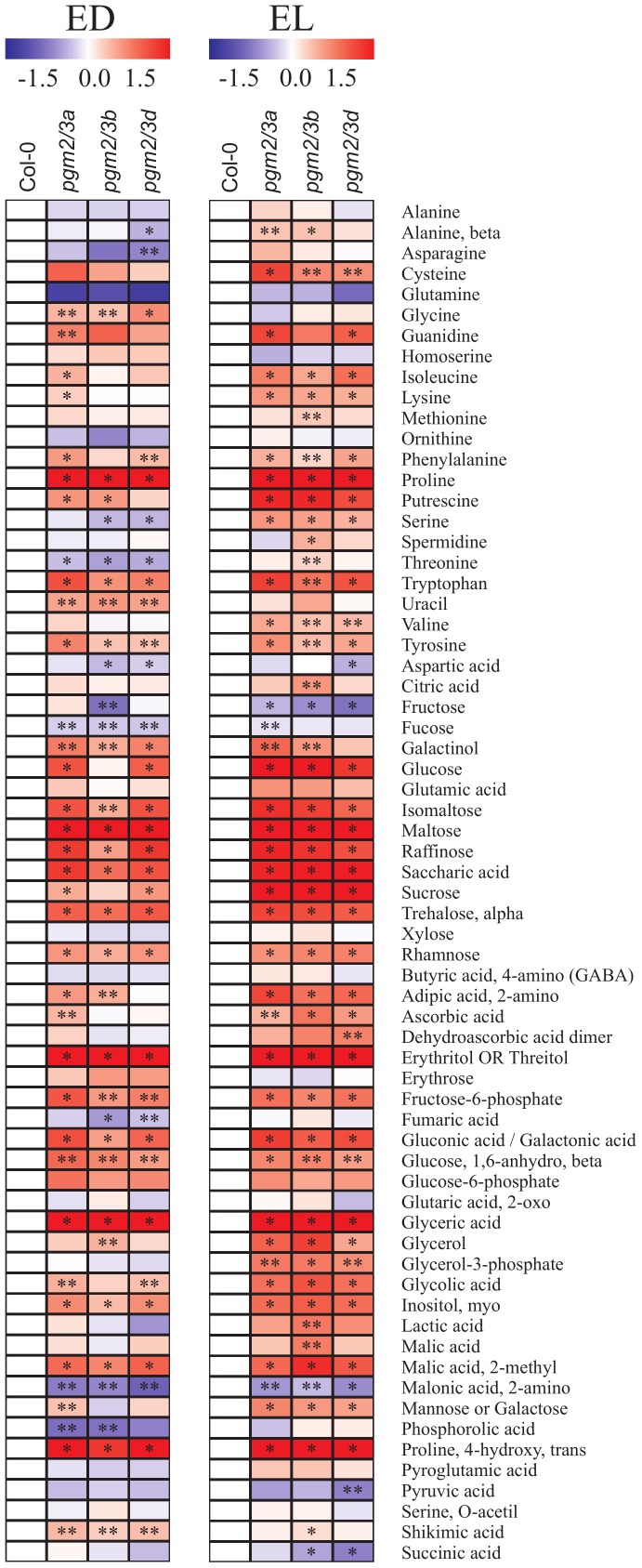
Overlay heat map of the metabolite changes in *pgm2/3* mutants in comparison with control (Co1-0) using false-color scale. Red or blue indicate that the metabolite content is increased or decreased, respectively. Five-week-old plants were grown under 12 h light/12 h dark conditions and harvested at the end of light phase (EL) and dark phase (ED), and three replicates represented 3–4 plants were analyzed (two technical replicates each); asterisks denote the significance levels as comparing *pgm2/3* mutants to Co1-0 : * *p*≤0.01;** *p*≤0.05.

Given that PGMs catalyze the interconversion of G1P and G6P, levels of sugar phosphates were determined. The *pgm2/3* plants displayed increased levels of G6P and fructose 6-phosphate (F6P) but G1P levels were similar to those in Col-0 (Fig. 2D–E). Nevertheless, further enzymes involved in the metabolism (DPE2 and phosphorylases) were not affected (Fig. S3D in File S1). In addition metabolic profiling was performed, revealing that numerous metabolites were increased both at the end of light and dark phase. At the end of the light period clear increases were seen in a range of sugars including maltose, glucose, trehalose, isomaltose and raffinose as well as the sugar alcohols galactinol, inositol and erythritol or threitol but fructose was unchanged or even decreased. Similarly, a large number of amino and organic acids were increased in the transgenic lines including tryptophan, proline, galacturonic acid, malate and shikimate (Fig. 3, Table S3 in File S1). By contrast, relatively few metabolites were consistently decreased in the transgenic lines at this time point those that were included were ornithine, phosphoric acid, asparagine, glutamine, and malonate. Consistent with these global effects on the primary metabolome being strongly influenced by the sugar status and more specifically by a likely inhibition of sucrose export, they became considerably stronger and more consistent by the end of the night. At this time point all three transgenic lines display alterations including maltose, glucose, trehalose, isomaltose, raffinose, galactinol, inositol, and erythritol or threitol, fructose 6-phosphates, tryptophan, proline, galacturonic acid, malate, and shikimate, which were also elevated in the day. Additionally, the levels of amino adipic acid, guanadine, glutamate, glycolate, lactate, and the branched chain amino acid increased in the dark. As for the situation observed in the light this is most likely the result of inhibition of sucrose export from the leaves. By contrast, at the end of the night the levels of malonate, pyruvate, glutamine and to a lesser extent succinate were significantly decreased in the transgenic lines. The exact reasons underlying these decreases are, however, unclear from the current study.

**Figure 4:**
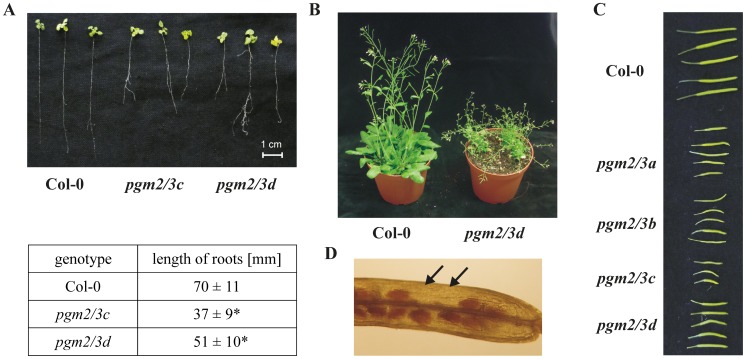
Roots and siliques of Col-0 and *pgm2/3* plants. A, Root length and morphology of Col-0 and *pgm2/3* lines. Plants were grown on vertical MS plates without any external sugar and antibiotics under long day conditions (16 h light/8 h dark). Plants were two-week-old. Length of central roots was measured. Values are means ± SD (n = 26−35). Asterisks indicate significant difference from Col-0 (Student Test, P≤0.01) B, Mature Col-0 and *pgm2/3d* plants. Col-0 and *pgm2/3* plants were six and 11- week-old, respectively. C, Morphology of siliques of Col-0 and *pgm2/3* lines. D, *pgm2/3d* silique. Siliques were destained in chloral hydrate solution (2.5 g in 1 mL distilled water). Black arrows indicate absence of seeds. C–D, Plants were grown under 14 h light/10 h dark regime.

As G1P is strictly connected with formation of UDP-glucose in the cytosol, which acts as a major substrate for synthesis of cell wall constituents [40], crystalline cellulose and matrix component were analyzed. The *pgm2/3* lines displayed increased amounts of cell wall matrix components and in two of the lines the crystalline cellulose amount was altered (Table 2). Additionally, samples of cell wall matrix were hydrolyzed and the monomer composition was analyzed using HPAEC-PAD. The transgenic lines were characterized by an increased amount of all analyzed monosaccharides and changes in the arabinose/galactose ratio in comparison to Col-0 (Fig. S3E in File S1). For analyses of the impact of cPGM on roots Col-0 and two *pgm2/3* lines were grown on vertical MS plates. *amiRNA pgm2/3* plants carry antibiotic resistance markers, kanamycin and hygromicin. However, it was reported that hygromycin is toxic even to resistant plants during long exposure, which may cause their abnormal development [41]. Indeed, when *pgm2/3* plants were grown in the presence of antibiotics, roots of *pgm2/3* transgenic lines were much shorter and more branched as compared to Col-0 cultivated without antibiotics (data not shown). To avoid such effects, Col-0 and *pgm2/3* seeds were sown on vertical MS plates without antibiotics. After two weeks plants were gently removed from plates and the length of main root was measured (Fig. 4A). Additionally, the lack of cytosolic PGM activity was confirmed in these plants using native PAGE. The root length of transgenic plants was increased on plates without antibiotics (compared to MS plates containing antibiotics), which confirmed that the antibiotics might affect the root growth of the transgenic plants. However, even without antibiotics the root length of transgenic plants was significantly decreased in comparison to Col-0 (Fig. 4A).

**Table 2:** Amount of crystalline cellulose and of cell wall matrix in Col-0 and *pgm2/3*.

genotype	crystalline cellulose [mg/g FW]	cell wall matrix [mg/g FW]
Col-0	5.17±0.42	4.73±0.01
*pgm2/3a*	6.24±0.11*	7.42±0.85*
*pgm2/3b*	5.80±0.06**	6.28±0.33*
*pgm2/3c*	5.43±0.24	6.63±0.58*

Plants were grown under 12 h light/12 h dark regime and harvested at the end of the light phase (six-week-old). Values are means of four replicates representing a mix of 7–10 plants ± SD. Asterisks denote the significance levels as comparing *pgm2/3* mutants to Co1-0 : * *p*≤0.01;** *p*≤0.05.

Furthermore, it was observed that *pgm2/3* lines were delayed in silique development, as compared to Col-0, independent of growth conditions (short day, long day) (Fig. 4B). The *pgm2/3* transgenic lines develop mature siliques approximately after 10–11 weeks under long day conditions (14 h light/10 h dark regime), whereas Col-0 achieves this after five to six weeks. Siliques from *pgm2/3* lines are much smaller (Fig. 4C) and possess a lower number of seeds compared to Col-0 (data not shown). In addition missing seeds were observed in the siliques of the transgenics (Fig. 4D).

### Impact of simultaneous reduction of cytosolic and plastidial phosphoglucomutase activities on Arabidopsis plants

**Figure 5:**
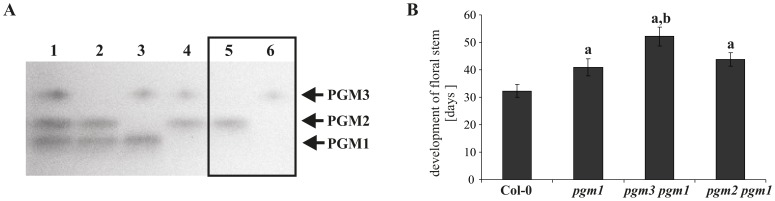
Characterization of knock-out mutants lacking one cytosolic and the plastidial PGM. A, Analysis of PGM activity in the Col-0 and *pgm3 pgm1* and *pgm2 pgm1* mutants using native PAGE and PGM activity staining. Separation gel 7.5% [T]. 35 µg proteins were loaded per lane. 1– Col-0, 2– *pgm3*, 3– *pgm2*, 4– *pgm1*, 5– *pgm3 pgm1*, 6– *pgm2 pgm1.* B, Analysis of floral stems development in Col-0 and different PGM knock-out plants. Plants were grown under long day conditions (14 h light/10 h dark). Days after germination were registered, when plants developed floral stems 1 cm long. Values are means ± SD (n = 24). a - significant difference from Col-0 (Student Test, *p*≤0.01), b - significant difference from *pgm1* (Student Test, *p*≤0.01).

Action of the plastidial phosphoglucomutase (PGM1) is an essential step in starch synthesis. Arabidopsis mutants lacking PGM1 are strongly reduced in starch content [1], [2]. In order to analyze the influence of single PGM2 or PGM3 mutation in the *pgm1* background, *pgm2* and *pgm3* mutants were crossed with *pgm1*. Both *pgm2 pgm1* and *pgm3 pgm1* are similar in growth compared to *pgm1,* under long day conditions (Fig. S4 in File S1). Crude extracts from double mutants were subjected to native PAGE and PGM activity staining (Fig. 5A). Both double mutants possess one band of cPGM activity each. Total PGM activity was reduced to 38±2% for *pgm3 pgm1* mutants and 36±2% for *pgm2 pgm1* plants (wt = 100%; n = 3).

Both double mutants possess very low yet still detectable amounts of starch (Table 3). *pgm3 pgm1* mutants revealed an elevated starch amount both in the light and in the dark compared to *pgm1*. However, when plants were grown under 12 h light/12 h dark or 16 h light/8 h dark, these results were not reproduced, as starch content was similar in *pgm1* and both double mutants under these photoperiod regimes (data not shown).

**Table 3:** Starch and soluble sugar content in Col-0 and PGM knock-out mutants.

genotype	starch content [mg glc equiv./g FW]		soluble sugars content (7 h in the light) [µmol/g FW]		
	7 h in the light	3.5 h in the dark	glucose	fructose	sucrose
Col-0	2.930±0.303	3.738±0.196	1.03±0.20	0.28±0.03	1.88±0.28
*pgm1*	0.012±0.003	0.010±0.001	4.23±0.65	1.04±0.21	2.69±0.11
*pgm3 pgm1*	0.025±0.005*	0.023±0.004*	4.91±0.59	0.94±0.04	2.70±0.17
*pgm2 pgm1*	0.015±0.003	0.016±0.003	4.67±0.51	0.87±0.11	2.74±0.31

Plants were grown under long day conditions (14 h light/10 h dark). Plants were five-week-old. Values are means of three biological replicates (two technical replicates each) ± SD. Asterisks indicate values significantly different from *pgm1* and *pgm2 pgm1* (Student Test, *p*≤0.05).

**Figure 6:**
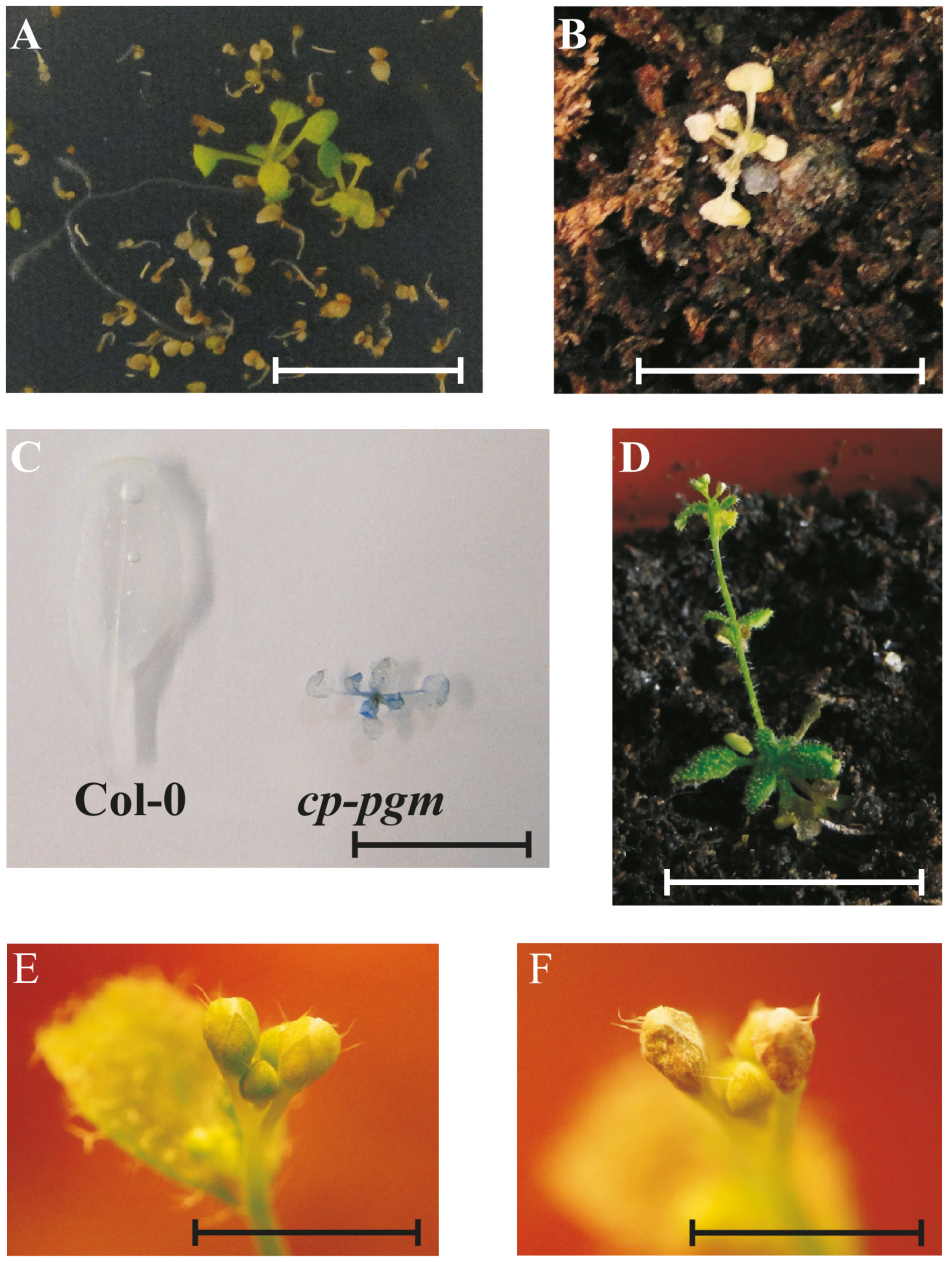
Growth phenotype of *cp-pgm* plants. A, Seeds were sowed on MS medium containing sucrose and antibiotics (kanamycin [50 µg/mL], hygromycin [50 µg/mL]). Plants were grown under long day conditions (16 h light/8 h dark) and were two-week-old. Bar = 1 cm. B, *cp-pgm* plant before trypan blue staining. C, Col-0 and *cp-pgm* plants after trypan blue staining. The *cp-pgm* plant was five- week-old, germinated on MS plate (as above) and the two last weeks grown under continuous illumination. Leave of Col-0 from three-week-old plant grown under 12 h light/12 h dark conditions. Bars = 1 cm. D–F, Phenotype of cp-*pgm* plants under continuous illumination. Seeds were germinated on MS medium containing sucrose with antibiotics (kanamycin [50 µg/mL], hygromycin [50 µg/mL]). After four weeks plants were transferred to soil and grown further under continuous illumination. D, Plant was six-week-old. Bar = 1 cm. E–F, Flower buds of *cp-pgm* transgenic plants. Plant was six-week-old (E) and seven-week-old (F). Bars = 1 mm.

Furthermore, *pgm1* and both double mutants displayed elevated levels of soluble sugar compared to Col-0 (Table 3). Additionally, it was consistently observed that the double knock-out mutants flowered significantly later compared to Col-0 (data not shown). Therefore, floral stem development was investigated. *pgm1* mutants were delayed in floral stem development compared to Col-0, which is consistent with a previous report [42]. The *pgm2 pgm1* mutant displayed a floral stem development time similar to that of *pgm1*, by contrast *pgm3 pgm1* plants were significantly delayed (Fig. 5B). Although, *pgm1, pgm2 pgm1*, and *pgm3 pgm1* plants contained very low amounts of starch, they were not strongly compromised in growth under long day conditions and were able to develop normal flowers and seeds. By contrast, plants with reduced cPGM activity are strongly diminished in growth and seed development (Fig. 4). Therefore, transgenic Arabidopsis lines with a substantial reduction of total PGM were generated by introducing the cPGM *amiRNA* construct into *pgm1* mutants by Agrobacterium mediated transformation (*cp-pgm* plants). Seeds were germinated on MS medium supplemented with sucrose and antibiotics and transformants with well developed leaves and roots were identified (Fig. 6A). It was noted that sucrose is essential for *cp*-*pgm* seed germination, as seeds sown on sucrose-free MS medium with appropriate antibiotics were not able to germinate.

In order to prove that the transgenic lines are strongly reduced in total PGM activity, protein crude extracts were subjected to native PAGE and PGM activity staining. The *cp-pgm* plants did not display any residual PGM activity (Fig. S5 in File S1). As a control the same crude extracts were used for phosphorylase activity staining, revealing activities comparable to Col-0 for both the cytosolic and plastidial phosphorylase isoforms (data not shown). After approximately three weeks *cp-pgm* plants were transferred to soil at different light/dark conditions: 12 h light/12 h dark, 14 h light/10 h dark and continuous illumination. Independent of growth conditions, plants were very tiny and rapidly became chlorotic and dry (Fig. 6B). However, under prolonged light conditions and continuous illuminations plants stayed green longer. Nevertheless, trypan blue which selectively stains dead tissue revealed that the plants are not longer vital (Fig. 6C; [37]). That said, some transgenic *cp-pgm* plants were even able to develop normal looking flowering buds under continuous illumination (Fig. 6D–E), but further development of flowers failed as buds shriveled within one week (Fig. 6F). Even if plants were supplied for the entire growth period with exogenous sugars (MS medium+sucrose) they failed to grow to maturity (data not shown). Thus, significant reduction of total PGM activity leads to a dramatic dwarf phenotype and inability to develop functional flowers and seeds. Therefore, *cp-pgm* plants showed a more severe phenotype compared with transgenic potato plants reduced in total PGM activity [24]. Additionally, the phenotype exhibited by the lack of total PGM activity was corroborated by crossing *pgm2/3d* with *pgm1* (named *pgm2/3d pgm1* plants) which displayed the same phenotype as *cp*-*pgm* plants (data not shown). Despite of the tiny amount of available leaf material, initial analysis of the starch content in *pgm2/3d pgm1* was performed revealing that *pgm2/3d pgm1* plants possess very low amounts of starch (0.21±0.02 µmol glc. equiv./g FW), similar to *pgm1* (0.25±0.06 µmol glc. equiv./g FW) at the middle of the day.

## Discussion

Analyses of single knock-out mutants of both cytosolic phosphoglucomutase isoforms (*pgm2* and *pgm3*) confirmed that the isoforms are redundant and expressed at a sufficient abundance to substitute for one another. Even the additional lack of PGM1 reveals only small alterations in metabolism and development in comparison to *pgm1* (Table 3, Fig. S4 in File S1). Furthermore, investigations with purified recombinant Arabidopsis enzymes, reveal that the kinetic properties of both cytosolic isoforms are very similar (for example the K_M'_s, using G1P as substrate, are PGM3 3.7±0.5 mM; PGM2 4.2±0.4 mM; [39]). The possible difference in substrate specificity observed for phosphoglucomutases of *Pseudomonas aeruginosa* [43] or *Giardia lamblia* [44], which show the additional interconversion of mannose 1-phosphate to mannose 6-phosphate, was not observed here. In competition experiments, where recombinant PGM2 or PGM3 were used with different amounts of mannose 1-phosphate in the presence of glucose 1-phosphate, no alteration in glucose 6-phosphate formation were observed. Furthermore, no formation of mannose 6-phosphate was detectable using HPAEC-PAD [39].

In contrast the *pgm2/3* lines reveal a very considerable phenotype. Even when the cytosolic phosphoglucomutase activity was below the detection limit, there was still a slight residual expression of both cytosolic isoforms (Fig. S3B in File S1). This is likely the reason for the severe yet not lethal phenotype. Thus, also the formation of seeds, albeit reduced or in some cases completely inhibited, could be explained and is in agreement with previous reports [24]. Furthermore, *pgm2/3* reveals alterations in cell wall composition, which were not previously detected in transgenic potato plants with strong reduction of cPGM [22].

Surprisingly in the *pgm2/3* lines a strong increase in sucrose, as well as the starch breakdown derived maltose, was observed. A significant increase in sucrose was additionally detected in the single knock-out line *pgm2* (Table 1). The formation of sucrose in the light is dependent on cPGM activity, as G1P is essential for the formation of UDPglucose via both routes of sucrose synthesis. However, several pathways for formation of G1P and thereby sucrose remain in the cytosol of *pgm2/3* plants: (i), the conversion via the mentioned residual cPGM activity in the plants, (ii), the formation of G1P in the night by the pathway of starch derived maltose, disproportionating enzyme 2, cytosolic heteroglycans, and the cytosolic phosphorylase [12], [13], (iii), the direct transport of G1P from the chloroplasts into the cytosol as demonstrated from isolated chloroplasts [1]. That said on the basis of our results flux through all of these routes can be anticipated to be relatively minor since formation via the starch degradation pathway is restricted to the night period, and it was shown that the G1P transport rate across the chloroplast membrane is minor in Arabidopsis in comparison to situation observed in potato [27]. Furthermore, it has been demonstrated that G1P that is taken up by the Arabidopsis chloroplast is directly converted into starch via ADPglucose pyrophosphorylase pathway, indicating that free G1P is immediately metabolized thus reducing the possibility of the G1P export [1]. It is possible that the observed elevation of the expression of PGM1 (Fig. S3B in File S1) in the transgenic lines is an effort to overcome this limitation. Additionally, preliminary experiments point to an increased G1P transport rate in *pgm2/3* plants compared to Col-0 (more than 20%) when measuring G1P uptake with isolated chloroplasts (data not shown).

However, it is not possible to explain the increase of sucrose in *pgm2/3* compared to Col-0 merely in terms of its rate of synthesis. It would seem more likely to be the consequence of the reduced sink capacity in the heterotrophic tissues and, therefore, a reduced export from the leaves of these lines. When sink capacity is reduced, feedback to the autotrophic tissues occurs culminating in the high starch and maltose levels observed in these lines. Moreover, metabolic profiling reveals a massive effect on the entire plant metabolism. Furthermore, taking into account the carbohydrate partitioning between sucrose and starch, the increase in both is not unexpected. Sucrose is catabolized either by sucrose synthase or invertase. It is proposed that invertase rather than sucrose synthase might be the dominant route for sucrose catabolism in *A. thaliana* [45]. Consequently, products of sucrose catabolism would enter the hexose phosphate pool as G6P or F6P but not as G1P. Thus, it would appear that cPGM is essential for G1P production.

A strong reduction of G1P is also anticipated to affect the entire nucleotide sugar metabolism [40], resulting in reduced growth and altered cell wall formation. As shown for *pgm2/3* the composition of the cell wall is altered and the root length is reduced. This phenotype was also observed for plants deficient in cytosolic invertase (*cinv1*) revealing reduced cell wall flexibility, inhibited root cell elongation and shorter roots [46]. Furthermore, mutants lacking two isoforms of cytosolic invertase (*cinv/cinv2*) are drastically reduced in root growth [45].

Additionally, a development of curly leaves was described in plants exhibiting reduced expression of SUT1 [47], [48] or plants expressed yeast derived invertase [49], [50], [51]. This leaf phenotype was postulated to be due to osmotic problems associated with carbohydrate accumulation, which is similar to the situation observed for *pgm2/3*. However, it is important to note that in some cases plants with alteration in cell wall synthesis, downstream of G1P, also display such curled leaves [52].

The tiny *cp-pgm* plants reveal an even more severe phenotype. Indeed under normal growth conditions these perturbations are lethal. Germination was only observed, when sucrose was supplemented, but also under these conditions complete formation of inflorescence and seeds were inhibited. As the expected residual cPGM activity is similar to the parental *pgm2/3* lines (not detectable), this is a strong indication that the glucose-phosphate interconversion via PGM1 and formation of G1P via the starch degradation pathway are essential in *pgm2/3* plants for the creation of the residual levels of G1P. The observed phenotype is much more severe than that observed for transgenic potato lines lacking both cPGM and pPGM activities [25]. The strongest reduced line was reported to have decreased leaf fresh weight of up to 33 percent. One explanation for the less distinct phenotype for potato is that in these plants a residual activity of both the pPGM and cPGM was still detectable (both 4%, [26]). However, also a second point is to mention, that the transport rate for G1P over the plastidial membranes seems to be much higher in potato compared to Arabidopsis [1], [27]. Thus, the possible bypass of the PGM lack via G1P transport is minor in Arabidopsis and therefore results in the observed more pronounced phenotype. Nevertheless, the higher transport rate of G1P observed for potato tuber is insufficient to completely overcome the limitations by lacking PGMs, especially in heterotrophic tissues, as the reduction in tuber fresh weight is far more pronounced with up to 75% reduction [25]. Overall, this points to a more flexible metabolism related to alternative carbon fluxes in potato then in Arabidopsis in respect to starch/sucrose turn-over.

## Supporting Information

File S1 **Supporting Information containing Tables S1–S3 and Figures S1–S5. Table S1.** Primers used for PCR and qPCR analysis. **Table S2**. Chlorophyll content of Col-0 and *pgm2/3* plants. **Table S3.** Values of the metabolic profiling used for the generation of the heat map. **Figure S1**. Phosphoglucomutase activity in Arabidopsis leaves. **Figure S2.** Analysis of single knock-out lines *pgm2* and *pgm3* and Col-0 under long day conditions (14 h light/10 h dark). **Figure S3.** Characterization of Col-0 and *pgm2/3* plants. **Figure S4.** Growth phenotypes of Col-0 and PGM knock-out mutants. **Figure S5.** Phosphoglucomutase activity in Col-0 and PGM transgenic plants.
(PDF)
