# Supplementary material for: Reduction of the Cytosolic Phosphoglucomutase in Arabidopsis Reveals Impact on Plant Growth, Seed and Root Development, and Carbohydrate Partitioning
Source: PLoS One. 2014 Nov 17;9(11):e112468. doi: 10.1371/journal.pone.0112468 (PMC4234415; doi:10.1371/journal.pone.0112468)
Supplement: File S1 — Supporting Information containing Tables S1–S3 and Figures S1–S5. Table S1. Primers used for PCR and qPCR analysis. Table S2. Chlorophyll content of Col-0 and pgm2/3 plants. Table S3. Values of the metabolic profiling used for the generation of the heat map. Figure S1. Phosphoglucomutase activity in Arabidopsis leaves. Figure S2. Analysis of single knock-out lines pgm2 and pgm3 and Col-0 under long day conditions (14 h light/10 h dark). Figure S3. Characterization of Col-0 and pgm2/3 plants. Figure S4. Growth phenotypes of Col-0 and PGM knock-out mutants. Figure S5. Phosphoglucomutase activity in Col-0 and PGM transgenic plants. (PDF) [file pone.0112468.s001.pdf]

Supporting Information

Reduction of the cytosolic phosphoglucomutase in Arabidopsis reveals impact on plant growth, seed and root development, and carbohydrate partitioning.

Irina Malinova, Hans-Henning Kunz, Saleh Alseekh, Karoline Herbst, Alisdair R. Fernie, Markus Gierth, and Joerg Fettke

Table S1. Primers used for PCR and qPCR analysis.

All primers are given in the 5' to 3' direction. Primer for generation of *amiRNA* lines were designed using http://wmd3.weigelworld.org tool and for qPCR using Quant Prime qPCR primer design tool [1].

Gene	Analysis	Primer sequence
At1g70730	*pgm2* mutant SALK_068481(AR)	TATTGGAGACAGCGTCTTCTG<br>TCGACAGGTTCGGATCATATC
At1g23190	*pgm3* mutant SALK_023069(AZ)	GCGACAGGTTGATATTTCTGC<br>TTGGCATGCTCCTAAAACAAG
At1g70730<br>At1g23190	*amiRNA*	Primer A:<br>CACCCTGCAAGGCGATTAAGTTGGGTAAC<br>Primer B:<br>GCGGATAACAATTTCACACAGGAAACAG<br>I miR-s:<br>gaTCTGTTAAGATAAATGCGCCTtctctcttttgtattcc<br>II miR-a:<br>gaAGGCGCATTTATCTTAACAGAtcaaagagaatcaatga<br>III miR*s:<br>gaAGACGCATTTATCATAACAGTtcacaggtcgtgatatg<br>IV miR*a:<br>gaACTGTTATGATAAATGCGTCTtctacatatatattcct
At1g70730	PGM2 expression	TGAAGACATTGTCCGCCAGCAC<br>CTGCAGTTGCGTCTACGTTCTC
At1g23190	PGM3 expression	AGGAAGCTTTGTCTCCTCTGGTTG<br>TCGGCCTGTGAACTCTTCCATC
At5g51820	PGM1 expression	ATTTCCGTGCTGGTCCTAAGGG<br>TCGGCAACACGATCAAGAGCAC
At3g18780	Actin2 expression	TCTTCCGCTCTTTCTTTCCAAGC<br>ACCATTGTCACACACGATTGGTTG

Table S2. Chlorophyll content of Col-0 and *pgm2/3* plants.

Plants were grown under 12 h light / 12 h dark conditions and harvested after five weeks. Chlorophyll was extracted from leaf material using 80 % [v/v] acetone and measured photometrically [2]. Values are means of four replicates represented mix of 8-12 plants ± SD. Asterisk indicates value significantly different from Col-0 (Student Test, $p \leq 0.05$).

genotype	chlorophyll content [mg / g FW]
Col-0	1.55 ± 0.13
pgm2/3a	1.73 ± 0.17
pgm2/3b	1.85 ± 0.19*
pgm2/3c	1.76 ± 0.20

Table S3. Values of the metabolic profiling used for the generation of the heat map.

Values are means of three replicates represented 3-4 plants (two technical replicates each) ±SD.

metabolite	end of the light phase				end of the dark phase			
	**Col-0**	***pgm2/3a***	***pgm2/3b***	***pgm2/3d***	**Col-0**	***pgm2/3a***	***pgm2/3b***	***pgm2/3d***
Alanine	4.281 ± 2.169	5.088 ± 2.565	4.574 ± 1.303	3.936 ± 1.228	4.158 ± 1.300	3.658 ± 1.352	3.614 ± 1.089	3.561 ± 1.277
Alanine, beta	0.038 ± 0.005	0.048 ± 0.010	0.048 ± 0.005	0.042 ± 0.005	0.031 ± 0.004	0.029 ± 0.003	0.030 ± 0.002	0.024 ± 0.002
Asparagine	0.127 ± 0.107	0.169 ± 0.108	0.139 ± 0.095	0.129 ± 0.084	0.137 ± 0.124	0.112 ± 0.064	0.081 ± 0.041	0.087 ± 0.036
Cysteine	0.005 ± 0.001	0.012 ± 0.005	0.009 ± 0.003	0.008 ± 0.003	0.005 ± 0.002	0.010 ± 0.005	0.008 ± 0.003	0.006 ± 0.001
Glutamine	1.999 ± 1.210	1.564 ± 0.855	1.532 ± 0.494	1.118 ± 0.413	1.656 ± 1.155	0.705 ± 0.360	0.773 ± 0.287	0.677 ± 0.189
Glycine	2.054 ± 0.429	1.717 ± 0.414	2.227 ± 0.129	2.270 ± 0.212	0.527 ± 0.119	0.709 ± 0.081	0.673 ± 0.076	0.850 ± 0.063
Guanidine	0.022 ± 0.011	0.051 ± 0.014	0.040 ± 0.018	0.045 ± 0.010	0.032 ± 0.006	0.055 ± 0.017	0.064 ± 0.054	0.047 ± 0.025
Homoserine	0.016 ± 0.003	0.012 ± 0.004	0.014 ± 0.002	0.014 ± 0.002	0.009 ± 0.002	0.011 ± 0.004	0.012 ± 0.002	0.011 ± 0.001
Isoleucine	0.325 ± 0.072	0.560 ± 0.080	0.466 ± 0.074	0.603 ± 0.081	0.278 ± 0.043	0.381 ± 0.042	0.291 ± 0.044	0.348 ± 0.078
Lysine	0.100 ± 0.013	0.155 ± 0.017	0.145 ± 0.021	0.140 ± 0.016	0.082 ± 0.014	0.100 ± 0.010	0.083 ± 0.010	0.083 ± 0.008
Methionine	0.071 ± 0.011	0.079 ± 0.011	0.088 ± 0.009	0.081 ± 0.007	0.049 ± 0.008	0.057 ± 0.004	0.052 ± 0.004	0.053 ± 0.003
Ornithine	0.040 ± 0.007	0.043 ± 0.003	0.039 ± 0.013	0.038 ± 0.005	0.035 ± 0.020	0.028 ± 0.004	0.022 ± 0.003	0.027 ± 0.007
Phenylalanine	0.283 ± 0.028	0.392 ± 0.031	0.333 ± 0.030	0.414 ± 0.041	0.232 ± 0.043	0.351 ± 0.030	0.271 ± 0.026	0.304 ± 0.038
Proline	2.369 ± 0.770	27.174 ± 6.995	33.883 ± 6.715	25.434 ± 4.731	2.237 ± 0.565	17.396 ± 5.885	12.806 ± 1.705	19.633 ± 1.295
Putrescine	0.042 ± 0.005	0.112 ± 0.011	0.112 ± 0.019	0.092 ± 0.006	0.050 ± 0.016	0.079 ± 0.004	0.077 ± 0.009	0.060 ± 0.004
Serine	5.796 ± 1.079	8.988 ± 1.213	8.565 ± 0.525	7.955 ± 0.795	3.733 ± 0.410	3.479 ± 0.181	2.939 ± 0.300	2.919 ± 0.287
Spermidine	0.166 ± 0.037	0.147 ± 0.024	0.229 ±0.021	0.195 ± 0.028	0.150 ± 0.048	0.141 ± 0.039	0.142 ± 0.021	0.155 ± 0.019
Threonine	2.111 ± 0.261	2.262 ± 0.156	2.500 ± 0.179	2.198 ± 0.133	1.657 ± 0.168	1.335 ± 0.053	1.187 ± 0.078	1.243 ± 0.046
Tryptophan	0.031 ± 0.014	0.071 ± 0.019	0.055 ± 0.014	0.064 ± 0.017	0.031 ± 0.009	0.066 ± 0.017	0.048 ± 0.009	0.053 ± 0.011
Uracil	0.009 ± 0.004	0.010 ± 0.003	0.013 ± 0.004	0.009 ± 0.003	0.009 ± 0.003	0.013 ± 0.002	0.014 ± 0.004	0.013 ± 0.001
Valine	0.365 ± 0.066	0.524 ± 0.077	0.469 ± 0.067	0.482 ± 0.100	0.294 ± 0.051	0.346 ± 0.042	0.286 ± 0.039	0.290 ± 0.062
Tyrosine	0.137 ± 0.025	0.219 ± 0.027	0.179 ± 0.027	0.198 ± 0.019	0.089 ± 0.012	0.149 ± 0.026	0.113 ± 0.013	0.113 ± 0.018
Aspartic acid	0.822 ± 0.108	0.731 ± 0.124	0.823 ± 0.032	0.627 ± 0.065	0.767 ± 0.049	0.699 ± 0.061	0.606 ± 0.046	0.654 ± 0.033
Citric acid	3.203 ± 0.503	3.947 ± 0.768	4.917 ± 1.043	3.745 ± 0.886	3.790 ± 0.441	4.353 ± 1.258	4.057 ± 0.907	4.133 ± 0.583
Fructose	1.391 ± 0.175	1.083 ± 0.101	0.923 ± 0.151	0.814 ± 0.105	1.316 ± 0.425	1.468 ± 0.221	0.761 ± 0.183	1.288 ± 0.431
Fucose	0.151 ± 0.011	0.138 ± 0.009	0.141 ± 0.005	0.139 ± 0.010	0.148 ± 0.018	0.127 ± 0.011	0.124 ± 0.008	0.123 ± 0.005
Galactinol	0.360 ± 0.097	0.695 ± 0.248	0.553 ± 0.081	0.457 ± 0.060	0.298 ± 0.063	0.520 ± 0.175	0.418 ± 0.101	0.501 ± 0.084
Glucose	0.791 ± 0.229	2.272 ± 0.374	2.259 ± 0.620	1.916 ± 0.216	0.861 ± 0.381	1.808 ± 0.111	0.901 ± 0.263	1.713 ± 0.436
Glutamic acid	3.997 ± 2.122	6.335 ± 2.918	6.037 ± 2.675	5.213 ± 2.309	3.169 ± 1.949	3.989 ± 2.022	3.231 ± 1.554	3.578 ± 1.326
Isomaltose	0.003 ±0.001	0.006 ± 0.001	0.006 ± 0.001	0.005 ± 0.001	0.003 ± 0.001	0.006 ± 0.002	0.004 ± 0.001	0.006 ± 0.001
Maltose	0.002 ± 0.000	0.019 ± 0.001	0.015 ± 0.001	0.016 ± 0.002	0.002 ± 0.000	0.019 ± 0.001	0.018 ± 0.002	0.016 ± 0.002
Raffinose	0.161 ± 0.050	0.433 ± 0.161	0.401 ± 0.058	0.346 ± 0.041	0.123 ± 0.013	0.296 ± 0.101	0.182 ± 0.031	0.302 ± 0.045
Saccharic acid	0.004 ± 0.001	0.010 ± 0.001	0.011 ± 0.002	0.011 ± 0.001	0.004 ± 0.001	0.010 ± 0.001	0.007 ± 0.002	0.009 ± 0.001
Sucrose	1.855 ± 0.375	6.328 ± 0.929	7.710 ± 0.553	7.097 ± 0.953	1.337 ± 0.185	1.886 ± 0.153	1.586 ± 0.258	2.057 ± 0.451

Trehalose, alpha,alpha	0.033 ± 0.005	0.076 ± 0.023	0.073 ± 0.007	0.067 ± 0.013	0.027 ± 0.009	0.055 ± 0.017	0.051 ± 0.011	0.056 ± 0.003
Xylose	0.014 ± 0.002	0.015 ± 0.001	0.016 ± 0.001	0.014 ± 0.002	0.013 ± 0.002	0.012 ± 0.001	0.011 ± 0.001	0.011 ± 0.001
Rhamnose	0.052 ± 0.004	0.081 ± 0.005	0.086 ± 0.005	0.087 ± 0.005	0.048 ± 0.004	0.074 ± 0.004	0.067 ± 0.005	0.074 ± 0.005
Butyric acid, 4-amino (GABA)	0.072 ± 0.006	0.079 ± 0.023	0.078 ± 0.007	0.067 ± 0.008	0.061 ± 0.007	0.054 ± 0.006	0.054 ± 0.007	0.055 ± 0.008
Adipic acid, 2-amino	0.008 ± 0.002	0.018 ± 0.003	0.015 ± 0.003	0.016 ± 0.002	0.008 ± 0.001	0.012 ± 0.001	0.011 ± 0.002	0.008 ± 0.002
Ascorbic acid	0.085 ± 0.018	0.116 ± 0.018	0.153 ± 0.040	0.130 ± 0.017	0.088 ± 0.019	0.118 ± 0.019	0.087 ± 0.014	0.091 ± 0.023
Dehydroascorbic acid dimer	0.301 ± 0.131	0.414 ± 0.102	0.505 ± 0.203	0.511 ± 0.113	0.398 ± 0.142	0.475 ± 0.156	0.366 ± 0.118	0.375 ± 0.110
Erythritol OR Threitol	0.016 ± 0.002	0.053 ± 0.005	0.056 ± 0.002	0.060 ± 0.005	0.014 ± 0.002	0.041 ± 0.003	0.041 ± 0.002	0.044 ± 0.002
Erythrose	0.008 ± 0.003	0.008 ± 0.002	0.007 ± 0.002	0.008 ± 0.003	0.005 ± 0.003	0.007 ± 0.002	0.008 ± 0.002	0.008 ± 0.002
Fructose-6-phosphate	0.006 ± 0.003	0.012 ±0.003	0.011 ± 0.003	0.012 ± 0.002	0.006 ± 0.002	0.012 ± 0.003	0.009 ± 0.002	0.010 ± 0.002
Fumaric acid	6.621 ± 1.659	6.491 ± 0.975	7.312 ± 1.055	6.210 ± 0.379	4.167 ± 0.694	3.580 ± 0.353	2.906 ± 0.264	3.400 ± 0.303
Gluconic acid / Galactonic acid	0.036 ± 0.005	0.086 ± 0.007	0.074 ± 0.004	0.079 ± 0.008	0.035 ± 0.009	0.077 ± 0.003	0.052 ± 0.007	0.069 ± 0.007
Glucose, 1,6-anhydro, beta	0.797 ± 0.178	1.318 ± 0.219	1.327 ± 0.387	1.186 ± 0.325	0.663 ± 0.215	1.266 ± 0.490	1.096 ± 0.281	1.004 ± 0.163
Glucose-6-phosphate	0.006 ± 0.003	0.010 ± 0.004	0.009 ± 0.003	0.009 ± 0.002	0.005 ± 0.003	0.010 ± 0.004	0.008 ± 0.002	0.009 ± 0.003
Glutaric acid, 2-oxo	0.014 ± 0.003	0.015 ± 0.003	0.016 ± 0.005	0.011 ± 0.003	0.010 ± 0.002	0.009 ± 0.002	0.011 ± 0.003	0.009 ± 0.002
Glyceric acid	0.133 ± 0.024	0.548 ± 0.064	0.603 ± 0.026	0.651 ± 0.047	0.047 ± 0.004	0.197 ± 0.011	0.155 ± 0.017	0.217 ± 0.010
Glycerol	0.153 ± 0.026	0.300 ± 0.094	0.355 ± 0.061	0.221 ± 0.030	0.167 ± 0.025	0.203 ± 0.057	0.229 ± 0.056	0.195 ± 0.021
Glycerol-3-phosphate	0.007 ± 0.002	0.012 ± 0.003	0.012 ± 0.003	0.011 ± 0.003	0.007 ±0.002	0.007 ± 0.003	0.007 ± 0.001	0.007 ± 0.001
Glycolic acid	0.016 ± 0.004	0.030 ± 0.006	0.034 ± 0.005	0.030 ± 0.003	0.012 ± 0.002	0.017 ± 0.004	0.015 ± 0.003	0.016 ± 0.003
Inositol, myo	0.425 ± 0.063	0.798 ± 0.073	0.850 ± 0.084	0.791 ± 0.039	0.423 ± 0.062	0.686 ± 0.073	0.555 ± 0.036	0.677 ± 0.038
Lactic acid	0.168 ± 0.035	0.247 ± 0.071	0.297 ± 0.046	0.269 ± 0.099	0.225 ± 0.074	0.254 ± 0.092	0.206 ± 0.142	0.155 ± 0.067
Malic acid	0.801 ± 0.228	1.015 ± 0.113	1.385 ± 0.240	0.995 ± 0.172	0.473 ± 0.103	0.538 ± 0.159	0.443 ± 0.074	0.575 ± 0.133
Malic acid, 2-methyl	0.020 ± 0.005	0.038 ± 0.003	0.051 ± 0.006	0.041 ± 0.005	0.017 ± 0.003	0.032 ± 0.003	0.028 ± 0.003	0.033 ± 0.004
Malonic acid, 2-amino	0.005 ± 0.001	0.003 ± 0.001	0.004 ± 0.000	0.003 ± 0.001	0.004 ± 0.001	0.002 ± 0.000	0.002 ± 0.000	0.002 ± 0.001
Mannose OR Galactose	0.047 ± 0.004	0.078 ± 0.009	0.071 ± 0.008	0.068 ± 0.004	0.045 ± 0.010	0.058 ± 0.006	0.039 ± 0.005	0.054 ± 0.008
Phosphoric acid	2.080 ± 0.527	1.697 ± 0.620	2.226 ± 0.679	2.254 ± 0.941	3.263 ± 1.091	1.845 ± 0.609	1.891 ± 0.509	2.040 ± 0.724
Proline, 4-hydroxy, trans	0.037 ± 0.007	0.145 ± 0.017	0.161 ± 0.017	0.150 ± 0.015	0.037 ± 0.003	0.112 ± 0.010	0.089 ± 0.007	0.118 ± 0.009
Pyroglutamic acid	7.052 ± 1.349	8.883 ± 2.446	8.933 ± 2.352	7.930 ± 1.941	7.376 ± 1.688	6.715 ± 1.549	6.263 ± 1.472	6.317 ± 1.785
Pyruvic acid	0.101 ± 0.031	0.072 ± 0.021	0.078 ± 0.009	0.063 ± 0.012	0.086 ± 0.016	0.068 ± 0.017	0.073 ± 0.013	0.069 ± 0.016
Serine, O-acetyl	0.018 ± 0.003	0.019 ± 0.004	0.018 ± 0.001	0.016 ± 0.002	0.016 ± 0.003	0.015 ± 0.003	0.017 ± 0.002	0.015 ± 0.001
Shikimic acid	0.259 ± 0.020	0.275 ± 0.044	0.295 ± 0.018	0.276 ± 0.033	0.181 ± 0.036	0.241 ± 0.041	0.222 ± 0.021	0.235 ± 0.013
Succinic acid	0.137 ± 0.025	0.122 ± 0.014	0.100 ± 0.005	0.085 ± 0.011	0.166 ± 0.032	0.172 ± 0.015	0.153 ± 0.018	0.132 ± 0.012

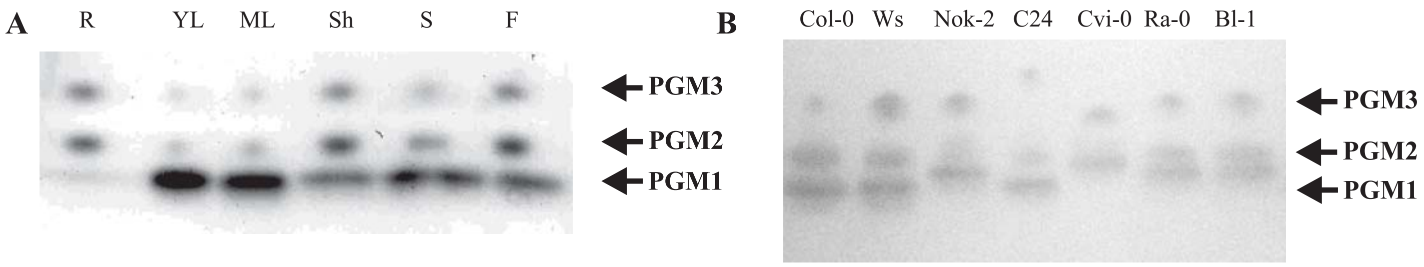

Figure S1. **Phosphoglucomutase activity in Arabidopsis leaves.**

A, PGM activity in different organs. 7 μg proteins per lane were loaded on the native gel (7.5 % [T]). Following electrophoresis, gels were incubated in PGM staining solutions (as described elsewhere [3]). Abbreviations: R - roots, YL - young leaves (≤8 mm), ML - mature leaves (≥30mm), Sh - shoots, S - siliques, F - flowers.

B, PGM activity in Arabidopsis accessions. Native PAGE followed by phosphoglucomutase activity staining. Gel 7.5 % [T]. 25 μg proteins were loaded per lane. Seeds of wild type *Arabidopsis thaliana* accessions (Nok-2, C24, Cha-0, Ra-0) were obtained from Prof. Dr. Altmann (IPK Gatersleben, Germany). Accessions Cvi-0 [N902] and Bl-1 [N968]) were ordered from Nottingham Arabidopsis Stock Centre (UK).

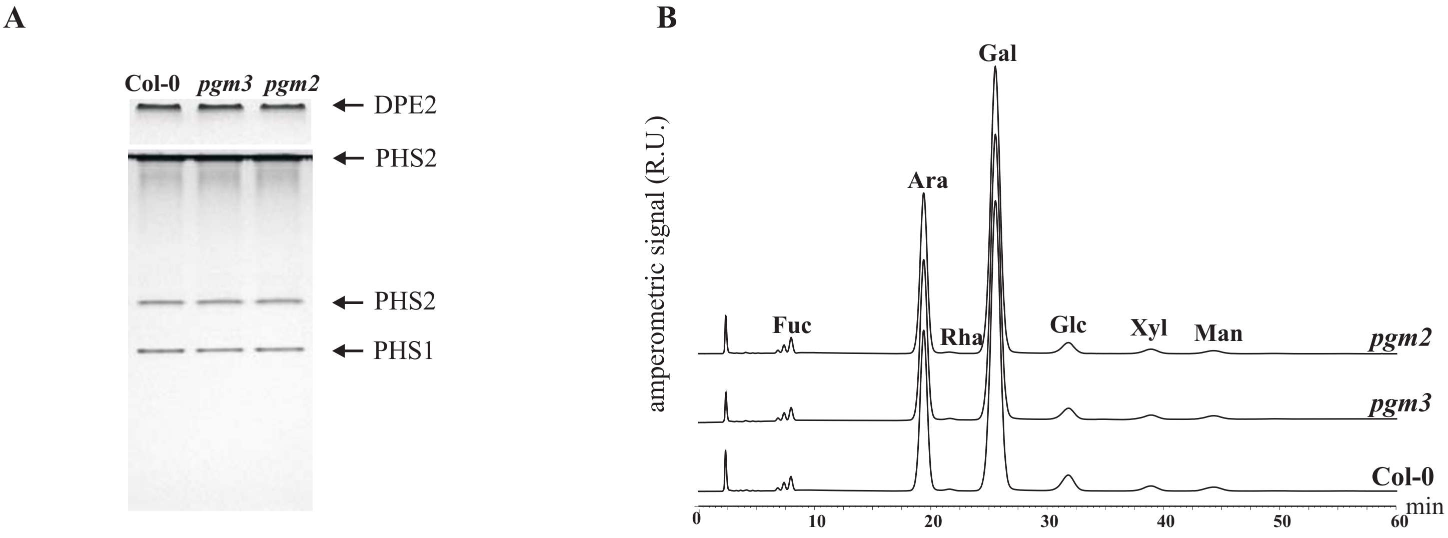

Figure S2. Analysis of single knock-out lines *pgm2* and *pgm3* and Col-0 under long day conditions (14 h light /10 h dark).

A, Activities of disproportionating enzyme 2 (DPE2) and phosphorylases. Protein crude extracts (7 µg) were subjected to native PAGE. Separation gel 7.5 % [T] contained 0.2 % [w/v] glycogen from oyster (Sigma). Following electrophoresis, separation gels were equilibrated in 100 mM citrate-NaOH pH 6.5. For phosphorylase activity staining separation gels were incubated in a mixture consisting of 100 mM citrate-NaOH and 20 mM sodium G1P (pH 6.5) overnight at room temperature (RT) and were then stained with iodine. DPE2 activity was visualized by incubating separation gels in 100 mM citrate-NaOH and 20 mM maltose (pH 6.5) overnight at RT prior to iodine staining.

B, Monomer composition of SHG_L isolated from Col-0, *pgm2,* and *pgm3* plants. Leaves were harvested at the end of the light phase. SHG_L were extracted and analyzed according to Fettke *et al*. [4]. 4 µg glucose equivalents were loaded on the HPAEC column. Signals are normalized to galactose. Two independent plant batches were analyzed and similar results were observed. Abbr.: Fuc - fucose, Ara - arabinose, Rha - rhamnose, Glc - glucose, Xyl - xylose, Man - mannose.

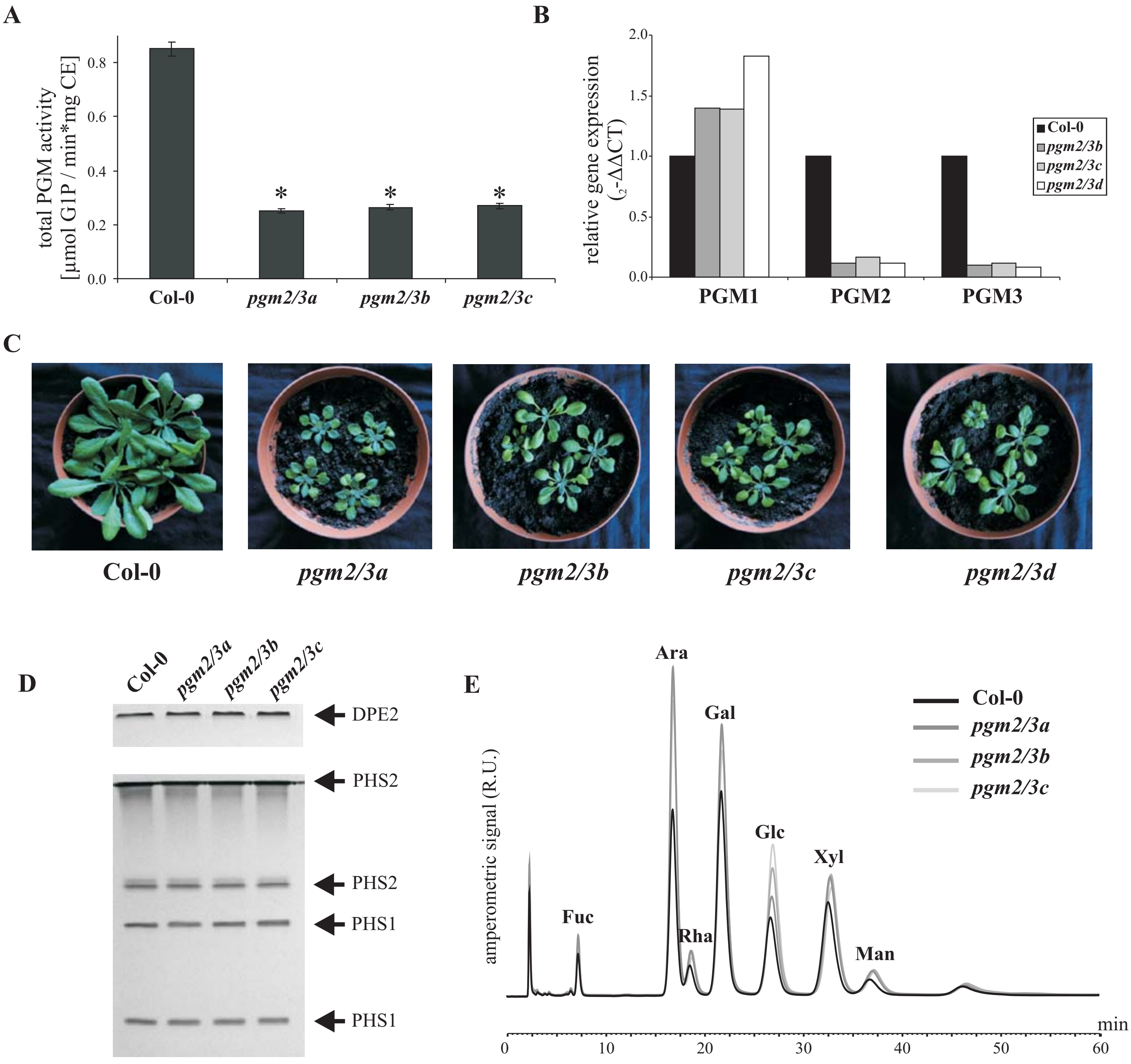

Figure S3. Characterization of Col-0 and *pgm2/3* plants.

A, Total PGM activity in leaf crude extracts (CE). PGM activity assay was performed as described by Fettke *et al.* (2008) with minor modification as measurement was started by adding of 17.5 mM G1P in reaction mixture. Values presented are means of two independent grown plant batches (two replicates each) ± SD. Asterisks indicate value significantly different from Col-0 (Student Test, $p \leq 0.01$).

B, Relative gene expression of PGM genes in Col-0 and *pgm2/3* lines. RNA was isolated from mixture of 5-10 plants using NucleoSpin RNA Plant (Macherey-Nagel). Synthesis of cDNA was performed using Maxima First Strand cDNA Synthesis Kit for RT-qPCR with dsDNase (Thermo Scientific) with oligo-dT primers. qPCR was carried out using the Fast SYBR Green Master Mix (Life Technologies) and Bio-Rad CFX Connect real-Time PCR System according to the supplier's recommendations. Values are means of three technical replicates derived from mix of at least seven plants. Primer sequences used are presented in Table S.1. PGM expression was normalized to the expression of Actin2. Relative gene expression was calculated according to Livak and Schmittgen [5].

Figure S3 continued

A-B, Plants were grown under 12 h light / 12 h dark conditions and plant rosettes were harvested in the middle of the light phase.

C, Growth phenotype of Col-0 and *pgm2/3* lines under long day conditions (14 h light / 10 h dark). Photographs were taken from five-week-old plants.

D, DPE2 and phosphorylases activity staining. Plants were grown in 12 h light / 12 h dark regime and harvested at the end of the dark phase. Native PAGE and incubation were performed as described in Fig. S2.

E, Monomeric composition of cell wall matrix. Samples were hydrolyzed with TFA (2 M final concentration). Equal fresh weight amounts were loaded on the HPAEC column. One replica of two is shown. Abbreviations as in Fig. S2.

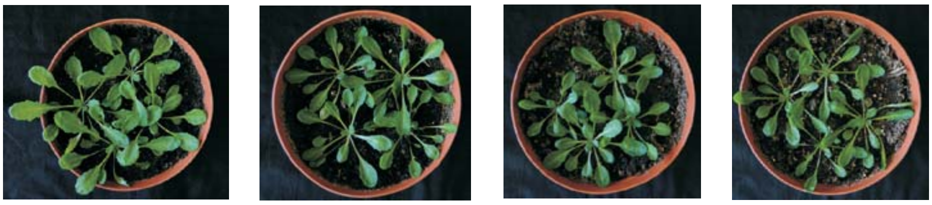

Col-0 ***pgm1*** ***pgm3 pgm1*** ***pgm2 pgm1***

Figure S4. Growth phenotypes of Col-0 and PGM knock-out mutants.
Plants were grown under long day conditions (14 h light / 10 h dark regime). The photographs were taken from five-week-old plants.

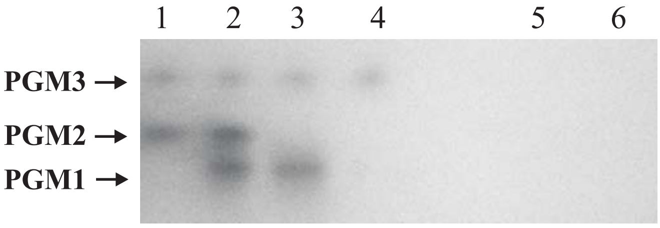

Figure S5. Phosphoglucomutase activity in Col-0 and PGM transgenic plants.
Native PAGE and phosphoglucomutase activity staining was performed as described in Fig. S1. Gel 7.5 % [T]. 8 µg of proteins were applied per lane. 1 - *pgm1*, 2 - Col-0, 3 - *pgm2*, 4 - *pgm2 pgm1*, 5 and 6 - *cp-pgm*. Col-0, *pgm1*, *pgm2, pgm2 pgm1* plants were six-week-old (12 h light /12 h dark regime). *cp-pgm* plants were four-week-old; plants were germinated and grown on MS plates containing sucrose and antibiotics (kanamycin [50 µg/mL], hygromycin [50 µg/mL]).

References (Supplementary Data)

1. Arvidsson S, Kwasniewski M, Riano-Pachon DM, Mueller-Roeber B. (2008) QuantPrime - a flexible tool for reliable high-throughput primer design for quantitative PCR. BMC Bioinformatics 9: 465.

2. Arnon DI.(1949) Copper enzymes in isolated chloroplasts: polyphenoloxidase in *Beta vulgaris*. Plant Physiol 24: 115.

3. Fettke J, Nunes-Nesi A, Alpers J, Szkop M, Fernie AR, et al. (2008) Alterations in cytosolic glucose-phosphate metabolism affect structural features and biochemical properties of starch-related heteroglycans. Plant Physiol 148:1614 -1629.

4. Fettke J, Eckermann N, Poeste S, Pauly M, Steup M. (2004) The glycan substrate of the cytosolic (Pho 2) phosphorylase isoform from *Pisum sativum* L.: identification, linkage analysis, and subcellular localization. Plant J 39: 933 - 946.

5. Livak KJ, Schmittgen TD. (2001) Analysis of relative gene expression data using real-time quantitative PCR and the $2^{-\Delta\Delta CT}$ method. Methods 25: 402 - 408.
